# Two novel *LHX3* mutations in patients with combined pituitary hormone deficiency including cervical rigidity and sensorineural hearing loss

**DOI:** 10.1186/s12902-017-0164-8

**Published:** 2017-03-16

**Authors:** Khushnooda Ramzan, Bassam Bin-Abbas, Lolwa Al-Jomaa, Rabab Allam, Mohammed Al-Owain, Faiqa Imtiaz

**Affiliations:** 1 Department of Genetics, King Faisal Specialist Hospital and Research Centre, P.O.Box 3354, Riyadh, 11211 Saudi Arabia; 2 Department of Pediatrics, King Faisal Specialist Hospital and Research Centre, Riyadh, Saudi Arabia; 3 Department of Medical Genetics, King Faisal Specialist Hospital and Research Centre, Riyadh, Saudi Arabia; 4 College of Medicine, Alfaisal University, Riyadh, Saudi Arabia

**Keywords:** *LHX3*, Pituitary hormone deficiency, Sensorineural hearing loss, Cysteine 146, Differential diagnosis

## Abstract

**Background:**

Congenital combined pituitary hormone deficiency (CPHD) is a rare heterogeneous group of conditions. CPHD-type 3 (CPHD3; MIM# 221750) is caused by recessive mutations in *LHX3*, a LIM-homeodomain transcription factor gene. The isoforms of LHX3 are critical for pituitary gland formation and specification of the anterior pituitary hormone-secreting cell types. They also play distinct roles in the development of neuroendocrine and auditory systems.

**Case presentation:**

Here, we summarize the clinical, endocrinological, radiological and molecular features of three patients from two unrelated families. Clinical evaluation revealed severe CPHD coupled with cervical vertebral malformations (rigid neck, scoliosis), mild developmental delay and moderate sensorineural hearing loss (SNHL). The patients were diagnosed with CPHD3 based on the array of hormone deficiencies and other associated syndromic symptoms, suggestive of targeted *LHX3* gene sequencing. A novel missense mutation c.437G > T (p. Cys146Phe) and a novel nonsense mutation c.466C > T (p. Arg156Ter), both in homozygous forms, were found. The altered Cys146 resides in the LIM2 domain of the encoded protein and is a phylogenetically conserved residue, which mediates LHX3 transcription factor binding with a zinc cation. The p. Arg156Ter is predicted to result in a severely truncated protein, lacking the DNA binding homeodomain.

**Conclusions:**

Considering genotype/phenotype correlation, we suggest that the presence of SNHL and limited neck rotation should be considered in the differential diagnosis of CPHD3 to facilitate molecular diagnosis. This report describes the first *LHX3* mutations from Saudi patients and highlights the importance of combining molecular diagnosis with the clinical findings. In addition, it also expands the knowledge of *LHX3*-related CPHD3 phenotype and the allelic spectrum for this gene.

## Background

Combined pituitary hormone deficiency (CPHD) refers to a rare heterogeneous group of conditions, which is characterized by true deficiency of at least two anterior pituitary hormones. CPHD has been linked with abnormalities in the genes encoding both signaling molecules and transcription factors, which have been shown to play a role in the development and maturation of the pituitary gland. These genes include *HESX1*, *LHX3*, *LHX4*, *POU1F1*, *PROP1*, *SIX6*, *OTX2*, *PTX2*, *GLI2* and *SOX3* [1–3]. The pituitary phenotype caused by genetic defects in these genes may be isolated (pure pituitary phenotype) or may be associated with variable extrapituitary maifestations (syndromic CPHD) [4]. Clinical presentation is variable, depending on the type and severity of deficiencies and at the age of diagnosis. If untreated, main symptoms usually include short stature, cognitive alterations or delayed puberty. CPHD is related to significant morbidity, especially hypoglycemia and its consequences in newborns. Treatment should be started immediately and a strict specialized follow-up is necessary [5]. Here, we describe the clinical, endocrinological, radiological and molecular features of three new cases of *LHX3*-related CPHD3.

Mutations in the *LHX3* gene (MIM# 600577), located on chromosome 9q34.3, underlie combined pituitary hormone deficiency type 3 (CPHD3, MIM# 221750). The encoded protein (Uniprot: Q9UBR4) is a LIM-homeodomain (LIM-HD) transcription factor, which features two amino-terminal LIM domains (cysteine-rich zinc-binding domain) and a centrally-located DNA-binding homeodomain [6]. In addition, the LHX3 protein also possesses a carboxyl terminus that includes the major trans-activation domain. Alternative splicing generates two isoforms, LHX3a and LHX3b, which are 397 and 402 amino acids long, respectively. These isoforms possess identical LIM domains, homeodomains and carboxyl termini but possess different amino-termini and differentially activate pituitary gene targets [7, 8].

Typically, genetically-confirmed CPHD3 patients with homozygous *LHX3* mutations present with hypopituitarism including deficiencies in the growth hormone (GH), thyroid stimulating hormone (TSH), prolactin (PRL), leutinizing hormone (LH), follicular stimulating hormone (FSH) and abnormal pituitary morphology. Adrenocorticotrophic hormone (ACTH) deficiency, cervical abnormalities with or without restricted neck rotation and sensorineural hearing loss (SNHL) has also been reported in a subset of these patients. The phenotypes in three patients (aged 2–11 year) in this report include CPHD, rigid cervical spine, restricted neck rotation, scoliosis, mild developmental delay and congenital hearing impairment. Targeted sequencing of the *LHX3* gene revealed two novel recessive variants in these patients. Finally, we surveyed the functional consequences of these mutations.

## Case presentation

We ascertained three patients with syndromic symptoms associated with CPHD. An informed written consent was used to recruit the patients and their family members. All affected patients underwent detailed examination at the departments of clinical genetics and pediatrics of King Faisal Specialist Hospital and Research Centre, Riyadh, Saudi Arabia. Laboratory testing, radiological investigation (X-ray) and audiologic assessments were performed. Pituitary examination was made by multisequential multiplanar magnetic resonance imaging (MRI) by applying standard scanning protocols [9]. The hormonal tests were measured by radioimmunoassay techniques by Roche Diagnostics, USA. Hormonal evaluation included basal levels for GH response to glucagon stimulation, TSH, PRL, LH, FSH, cortisol, ACTH and free thyroxine (FT4) levels. Growth biochemical markers including insulin growth factor 1 (IGF1) and insulin growth factor binding protein 3 (IGFBP3) were also assessed. The diagnoses of hormonal deficiencies were established using the normal range as cutoff limits: Basal GH (>10 μg/L), TSH (0.27–4.2 mU/L), PRL (2.5–15 μg/L), LH (0.1–3.3 U/L), FSH (0.1–7 U/L), ACTH (5–60 ng/L), cortisol (190–750 nmol/L), FT4 (12–22 pmol/L), IGF1 (115–498 ng/L) and IGFBP3 (0.7–3.6 mg/L).

### Cases

#### Patient 1, Family 1

This boy (II:2, Fig. 1a) was a product of an uneventful pregnancy and normal vaginal delivery at 39 weeks of gestation, whose healthy consanguineous parents had previously lost a daughter in infancy probably because of the same condition. At birth, he was noted to have a micropenis with a stretched penile length of 1.8 cm and undescended testes. Other dysmorphic features included short webbed neck with limited rotation and triangular face. During hypoglycemic attack at the 3^rd^ month of the life, ACTH and GH deficiencies were diagnosed. Later TSH and gonadotropin deficiencies were also found (see Table 1). Both TSH and FT4 were low suggesting central hypothyroidism. MRI of pituitary gland showed a small cystic lesion replacing the adenohypophysis of the pituitary gland representing developmental cystic abnormality. The pituitary stalk was present with normally placed neurohypophysis. Skeletal survey showed closure of sagittal suture causing dolichocephaly and hypoplasia of the facial bones. Thyroxine (25 μg daily), hydrocortisone (2.5 mg twice daily) and subcutaneous injections for GH (0.035 mg/kg/day) were initiated to normalize glycemia and to improve somatic growth at the 5^th^ month of age. His height was 107 cm at age of 7 years (on the 5th percentile, −2SD). Testosterone therapy was given at a dose of 25 mg monthly for 3 months, which improved penile length to 3.1 cm. He also had moderate SNHL at the age of 2 years.

**Fig. 1:**
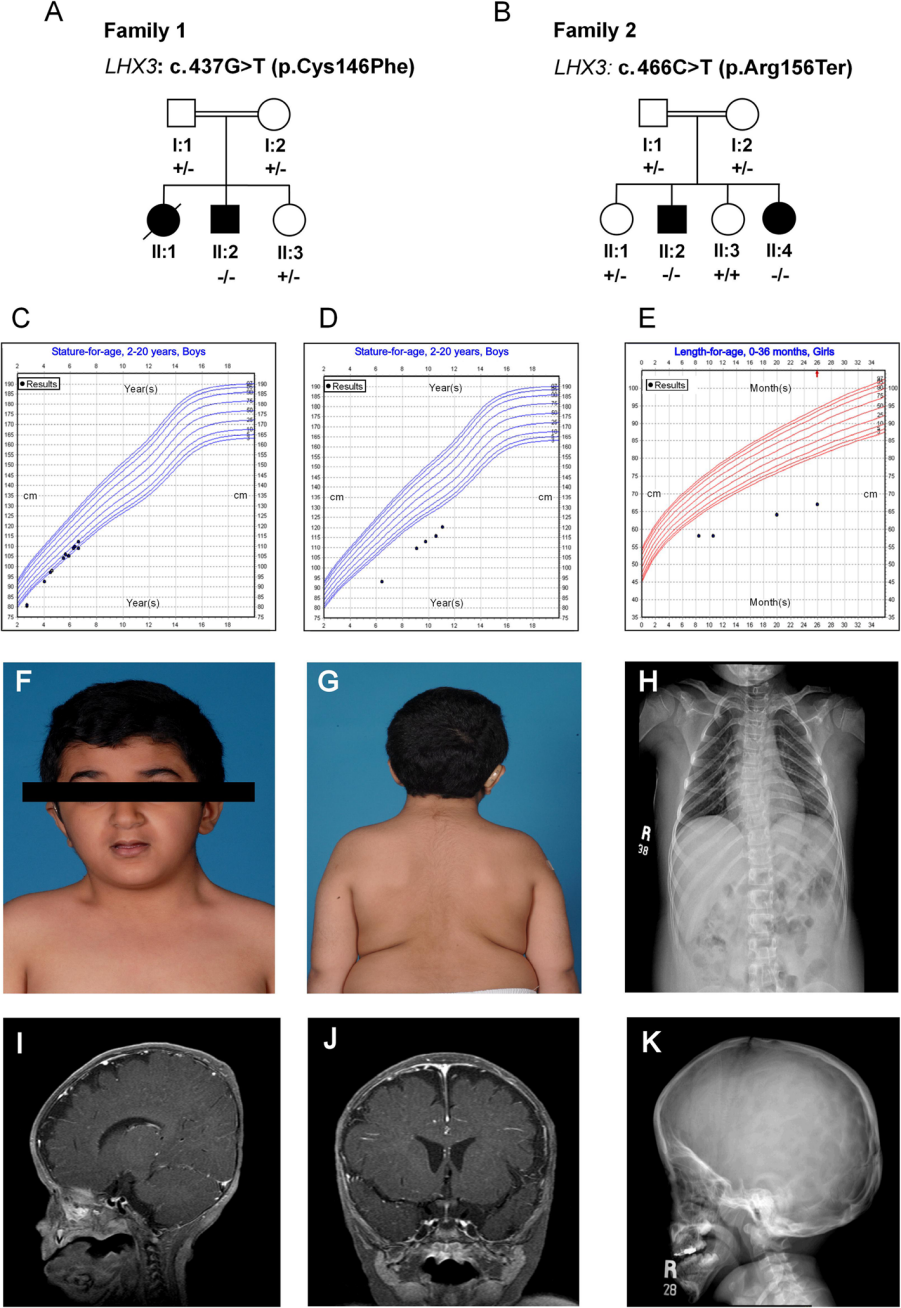
Family pedigrees, genotypes, growth charts for patients and clinical presentation. **a**, **b** Pedigrees of the families studied with CPHD3 demonstrating the recessive inheritance pattern. *Filled symbols* indicate affected individuals. The respective genotype is indicated below each individual. Symbols are: + for wild type allele;—for mutated allele. **c**, **d**, **e** Representative growth chart for Patients II:2 (Family 1), II:2 (Family 2) and II:4 (Family 2) showing reduced growth velocity for all patients and reduced response for patient 2 and 3 compared to patient 1. Affected patient 2 (II:2 from family 2) showing **f** neck rotation, **g** scoliosis and **h** lower thoracic scoliosis by spine x-ray. **i**, **j** Multisequential multiplanar brain MRI reveals pituitary gland hypoplasia for patient II:2 from family 1 **i** coronal view **j** sagittal view **k** skull x-ray demonstrates an increased anterior-posterior diameter of calvarium suggestive of dolicocephaly

**Table 1:** Clinical data for three patients with novel *LHX3* mutations

	Patient 1 (II:2, Family 1)	Patient 2 (II:2, Family 2)	Patient 3 (II:4, Family 2)
Age, years	7	11	2
Gestational age, weeks	39	39	39
Birth weight, kg (SD)	3.2 (0SD)	3 (− 0.7SD)	2.9 (− 0.7SD)
Birth length, cm (SD)	45 (−2.5SD)	NA	46 (−1.8SD)
Age of onset, months	3	2	2
Initial manifestation hypopituitarism	+	+	+
Basal GH μg/L (>10)	<0.2	<0.5	<0.5
TSH mU/L (0.27–4.2)	0.02	0.04	0.4
PRL μg/L (2.5–15)	0.1	0.1	NA
LH U/L (0.1–3.3)	<0.1	<0.1	<0.1
FSH U/L (0.1–7)	<0.1	<0.1	<0.1
ACTH ng/L (5–60)	6	8	16
cortisol nmol/L (190–750)	70	62	102
FT4 pmol/L (12–22)	4.4	9.3	10.3
IGF1 ng/L (115–498)	<25	<25	NA
IGFBP3 mg/L (0.7–3.6)	<0.5	<0.3	NA
Hypolplastic pituitary gland	+	+	-
Limited neck rotation	+	+	-
SNHL	+	+	+
Developmental delay (Mild)	+	+	+
Other findings	Dolichocephaly, Hypolasia of facial bones, frontal bossing, short webbed neck	Dolichocephaly, Thoracic scoliosis, squint	None
Mutation Identified	c.437G > T (p. Cys146Phe) Homozygous	c.466C > T (p. Arg156Ter) Homozygous	
Type of mutation	Missense mutation	Nonsense	
Effect on protein	Location a well-established domain	Null mutation	
Computation (in silico) predictive analysis	“Damaging”	“Damaging”	
Population data	Not annotated as polymorphism	Not annotated as polymorphism	
Functional data	Located in functional domain	Located in functional domain	
Allelic and family segregation data	Recessive mutation and strong segregation	Recessive mutation and strong segregation	
Other Evidence	Relevant to patient’s phenotype	Relevant to patient’s phenotype	
Variant classification (ACMG)	Likely pathogenic	Pathogenic	

*NA* not available

#### Patient 2, Family 2

The proband (II:2, Fig. 1b) of family 2 is the second child of healthy first degree parents, born via normal vaginal delivery at 39 weeks of gestation. He was initially evaluated in a local hospital for mild hypoglycemic attacks which were associated with jitteriness and irritability. He was first seen at our clinic at age of 2 years. He was clinically normal, apart from an abnormal head tilt secondary to abnormal neck posturing. His height was -3SD and he had a stretched penile length of 0.5 cm (−5.3 SD) with non-palpable testes. The patient had craniosynostosis causing plagiocephaly. Hypopituitarism was confirmed (Table 1). An MRI of his pituitary gland showed a small rudimentary adenohypophysis and a normal pituitary stalk present with normally placed neurohypophysis. A skeletal survey showed closure of sagittal suture causing dolichocephaly and thoracic scoliosis. L-thyroxine (50 mcg daily), hydrocortisone (2.5 mg twice daily) and GH injections (0.035 mg/kg/day) were initiated. Orchipexy was performed at the age of 2.5 years. Testosterone therapy (25 mg monthly) was given for 3 months which improved penile length to 1.5 cm. Two other testosterone courses were given at the age of 5 years and 10 years with a partial improvement in penile length to 3 cm. Bilateral moderate SNHL was also found at the age of 3 years. His growth improved in response to GH treatment; he had a growth velocity of 3–4 cm per year. His height was 110 cm at the age of 10 years (−4SD). The prescribed dose for GH injections was 0.035 mg/kg/day; however the patient was not compliant with treatment, which negatively affected the growth velocity and the ultimate height at the age of 10 years.

#### Patient 3, Family 2

The younger sister (II:4, Fig. 1b) of the patient 2 was born via normal vaginal delivery at 39 weeks of gestation. Her birth weight (2.9 kg) and birth length (46 cm) was adequate for gestational age. She also presented with jitteriness secondary to hypoglycemia at 2^nd^ month of age and was suspected to have hypopituitarism (Table 1), because her older brother had been diagnosed with the same condition before. Treatment thyroxine, hydrocortisone and GH injections were started. She has moderate SNHL and a normal skeletal survey. Magnetic resonance imaging showed a normal pituitary gland. Clinical features and hormonal levels for all three affected patients are summarized in Table 1.

### Genetic analyses

Genomic DNA extraction was carried out using PUREGENE DNA Extraction Kit according to manufacturer’s instructions (Gentra Systems, Minneapolis, MN) from the venous blood samples collected from each subject. All exons and adjacent exon-intron boundaries of the both *LHX3* transcripts were amplified in Veriti® 96-Well Fast Thermal Cycler (Applied Biosystems, Foster City, CA) using HotStar Taq DNA Polymerase (Qiagen, Germantown, MD). Primers were designed by the use of Primer3 web based server (http://frodo.wi.mit.edu/primer3/; sequences of primers used for PCR amplification are listed in Table 2). The PCR products were sequenced by dye termination sequencing using BigDye® Terminator Cycle Sequencing v3.1 Kit and ABI Prism 3730 Genetic Analyzer (Applied Biosystems, Inc., Foster City, CA, USA). Sequence analysis was performed using the SeqMan 6.1 module of the Lasergene (DNA Star Inc. WI, USA) software package and the results were compared with reference sequence. Accession numbers: Nucleotide and amino acid numbering are for *LHX3*, variant 2 (also known as isoform b) and correspond to NCBI reference sequence accession number NM_014564.3 for the cDNA and NP_055379.1 for the protein.

**Table 2:** Sequences of oligonucleotide primers used for PCR amplification

Primer	Sense Strand	Antisense Strand
*LHX3*_Exon 1a	5′- CAACCCAGCCAGGGAG - 3′	5′- GTTTCCATCTCTGTGTCCCG - 3′
*LHX3*_ Exon 1b	5′- CCCGGAGTCGCTTGGAC - 3′	5′- GCCCAGATCCTCTAGCTCC - 3′
*LHX3*_ Exon 2	5′- CAGCCCTGAGTCCTGTGG - 3′	5′- TGATTGTGAGGGGAGGAGTC - 3′
*LHX3*_ Exon 3	5′- CGGACAGAGCCTTCCTC - 3′	5′- GGAGAGAATTTCCCCGGAC - 3′
*LHX3*_ Exon 4 + 5	5′- CTTCCGAGAAGCCTGTG - 3′	5′- TCCATGGGAAATTCAGATCC - 3′
*LHX3*_ Exon 6	5′- CTGCAGGATGGGACTCTG - 3′	5′- CACCAGCCCTCCCTTGAC - 3′

### In silico prediction and pathogenicity assessment

For interpretation of the identified sequence variants as per ACMG guidelines, PolyPhen2 (Polymorphism phenotyping; http://genetics.bwh.harvard.edu/pph2/), SIFT (Sorting Intolerant From Tolerant; http://sift.jcvi.org/) and MutationTaster (http://www.mutationtaster.org/) were used for pathogenicity assessment [10]. Project HOPE ([11]; http://www.cmbi.ru.nl/hope/) was used to predict the possible structural changes in the mutant protein using a normal LHX3 structure (PDB-file: 2JTN). The sequences form the *Homo Sapiens* LHX3 protein and homologous proteins from other eukaryotic species were obtained from the National Center for Biotechnology Information (NCBI; http://www.ncbi.nlm.nih.gov/). A multiple alignment between these proteins was performed using Clustal Omega (http://www.ebi.ac.uk/Tools/msa/clustalo/).

## Results

**Fig. 2:**
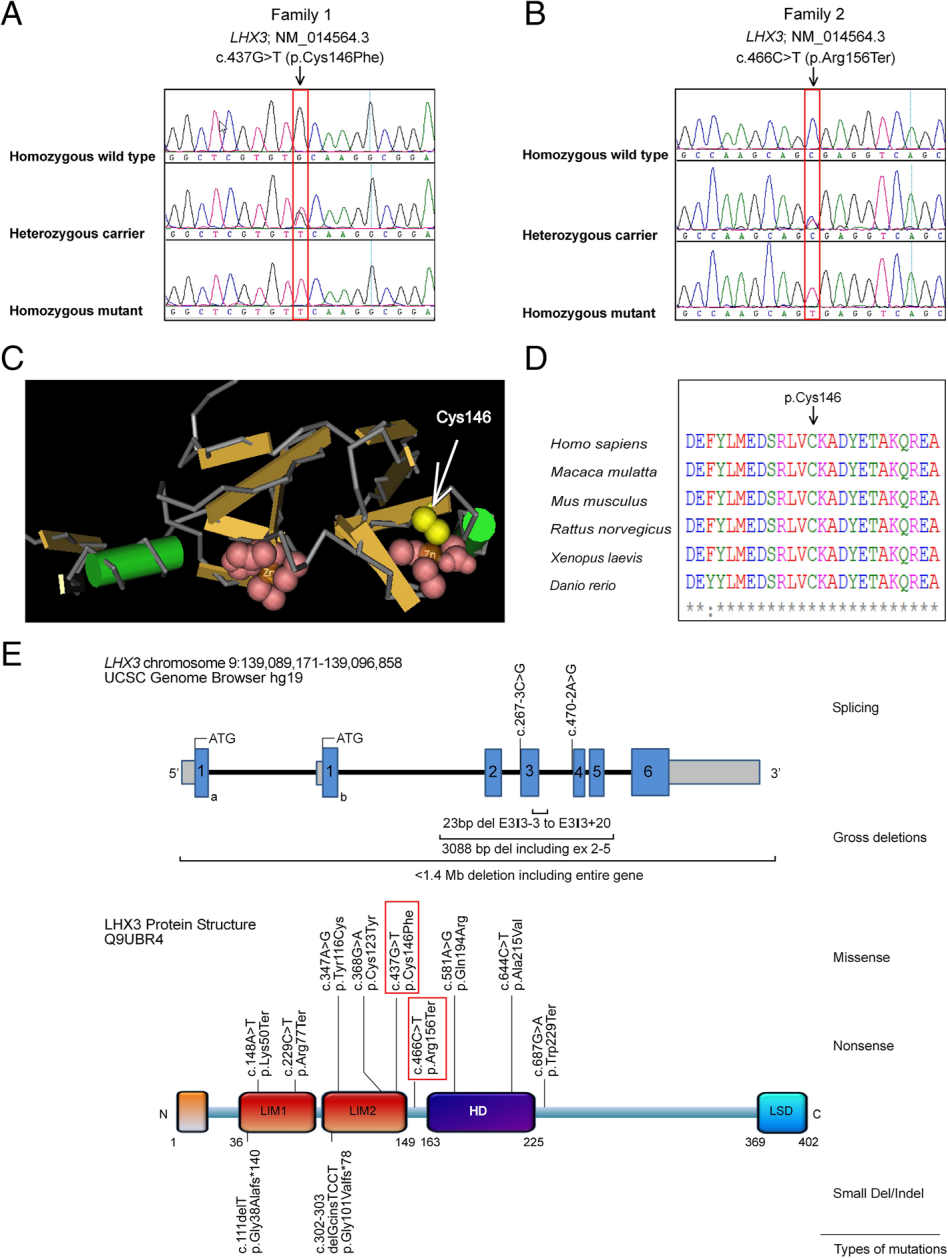
Identification of two novel mutations in *LHX3*. **a** Sequencing chromatogram indicating the homozygous wild type, heterozygous carrier and homozygous mutant forms. Homozygous c.437 G > T (p. Cys146Phe) mutation is identified in affected individual (II:2, family 1). **b** Homozygous c.466 C > T (p. Arg156Ter) mutation is identified in affected individuals (II:2 & II:4, family 2). Nucleotide and amino acid numbering correspond to NM_014564.3 for the cDNA and NP_055379.1 for the protein. Nucleotides were numbered using A of the ATG translation initiation codon as +1 nucleotide of the coding sequence. Mutations are highlighted (*arrow*). **c** Ribbon/Cartoon-presentation of zinc finger motif of the LHX3 consisting of α-helix (*green*) and β-sheets (*brown*). The Zn binding residues of LIM domains are highly conserved; cysteine at position 146 (*yellow*) is a zinc ligating residue, involved in binding with the zinc cation (brown). **d** The multiple-sequence alignment was generated with the Clustal Omega Multiple Sequence Alignment tool and depicts conservation of the crucial p. Cys146 residue during evolution. *Asterisk* (*) indicates positions which have a single, fully conserved residue. *Colon* (:) indicates conservation between groups of strongly similar properties - scoring > 0.5. **e** *LHX3* mutations associated with combined pituitary hormone deficiency. Schematic representation of intron-exon structure of *LHX3* gene, domain graph of the encoded protein (Uniprot identifier: Q9UBR4), and the genetic variants. Exons are designated as *boxes* 1–6 and introns are shown by *thin lines*. A full-length wild type LHX3 protein is shown, with its N-terminus and C-terminus. Alternative splicing generates two isoforms, LHX3a and LHX3b, which are 397 and 402 amino acids long respectively. The isoforms differ only in the amino-terminal domains. Other known functional domains are following: LIM domains (LIM); homeodomain (HD), and carboxyl trans-activation domain (LSD). Novel variants identified in our study are *boxed in red* alongside previously reported variants in HGMD database [28]. The mutations are grouped according to canonical classes and further identified by their amino acid changes

Sanger sequencing of *LHX3* gene in the affected boy (II:2 from pedigree, Fig. 1a) from the first consanguineous family led us to the identification of a homozygous nucleotide change (c.437G > T), resulting in a cysteine-to-phenylalanine substitution at 146 amino acid of the encoded protein (p. Cys146Phe) (Fig. 2a). In the two sibs (II:2 & II:4 from pedigree, Fig. 1b) from the second family, we identified homozygosity for a single base exchange (c.466C > T). At the protein level, this transition predicts change of amino acid 156 from arginine to a stop codon (p. Arg156Ter) (Fig. 2b). Parents and siblings of the affected patients were all healthy and show no signs or symptoms related to the disorder. In addition, they were either heterozygous or wild type for the identified mutations (Fig. 1a and b). p. Cys146Phe is predicted to be “damaging” based on PolyPhen2, SIFT and MutationTaster. p. Arg156Ter is also predicted to be “disease causing” by MutationTaster. Both the alterations were not annotated in either dbSNP [12], the 1000 Genomes Project database [13], or Exome Aggregation Consortium (ExAC) [14] and were absent in 400 normal chromosomes of ethnically matched controls, precluding that these represented benign or more common polymorphisms.

The identified p. Cys146Phe substitution resides in the LIM2 domain of the LHX3 protein (Q9UBR4). LIM domain is recognized as a tandem zinc-finger (Znf) structure that functions as a modular protein-binding interface and LIM homeodomain proteins have been shown to play roles in cytoskeletal organization, organ development, cell adhesion, cell motility and signal transduction [15, 16]. Znf motif mostly contain a pattern of cysteine and histidine residues that coordinately bind to zinc ions at the base of each of the two ‘fingers’, but in some cases they bind other metals such as iron, or no metal at all [17]. The cysteine at position 146 is a zinc ligating residue, involved in binding with the zinc cation (Fig. 2c). A three-dimensional-structure prediction analysis for p. Cys146Phe mutation by project HOPE predicts that the mutant residue (phenylalanine) will probably not fit in place of the smaller size wild-type cysteine residue which is buried in the core of the protein and will thus disturb the possible rearrangements of surrounding residues. In addition, the replacement of cysteine at p.146 will disrupt the putative interaction with zinc cation, thus affecting biological activity of LHX3. This cysteine is entirely conserved in LHX3 proteins in homologous sequences (Fig. 2d). It is part of the amino acid sequence ‘signature’ that defines the LIM domains proteins [18], indicating that the Cys146 residue is critical to overall LHX3 function. The identified nonsense mutation p. Arg156Ter is predicted to result in a premature stop codon, loss of entire DNA-binding homeodomain and carboxyl terminus causing the production of short, inactive LHX3 protein or non-sense mediated decay of the aberrant mRNA. Mutant LHX3 proteins lacking a homeodomain do not bind DNA and thus do not induce transcription from pituitary genes. A complete loss of function is assumed with this homozygous mutation.

Hence, the factors that p. Arg156Ter being a null variant and critical location of p. Cys146Phe in a well-established domain, their absence in population data/controls, computational evidence, segregation analysis and relevance to the patients phenotype, led us to classify these allelic variants; p. Arg156Ter and p. Cys146Phe as “pathogenic” and “likely pathogenic” respectively, according to the recommendations of ACMG guidelines [10] for the interpretation of sequence variants (see Table 1).

## Discussion and conclusions

**Fig. 3:**
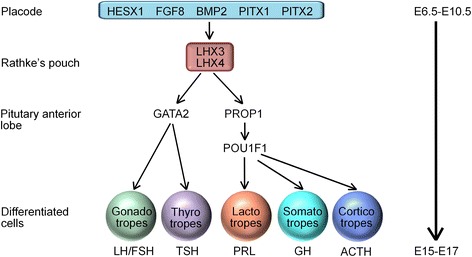
A simplified scheme of the development cascade representing the main transcription factors expression during pituitary development (adapted from de Moraes et al. [20]). Pituitary cell lineages are determined by the activation or repression of each transcription factor. LHX3 participates in the pituitary cell differentiation and maturation process. The anterior pituitary consists of five distinct cell types. These cells and their specific hormones are lactotropes, which produce PRL; somatotropes, which produce GH; gonadotropes, which produce LH and FSH; corticotropes, which produce ACTH; and thyrotropes, which produce TSH. Any mutation that alters the length, quality or quantity of any gene involved in the development cascade will result in pituitary development failure

Pituitary organogenesis is a highly complex process and tightly regulated cascade of several transcription activators and repressors, and signaling molecules [19, 20]. Anterior pituitary ontogenesis begins early around embryonic day (E) 7.5, corresponding to the first visualization of the pituitary placode. Briefly, during the early stage of pituitary development, which corresponds to E6.5-10.5 in mice, the extrinsic signaling pathways are activated, including bone morphogenetic protein (BMP2, BMP4), fibroblast growth factor (FGF8, 10 and 18), Sonic Hedgehog (SHH) and wingless (WNT4) pathways [21]. Within the developing Rathke’s pouch and anterior pituitary, a number of transcription factors are involved in a timely manner during the steps of differentiation; early acting such as paired homeodomain transcription factors (HESX1, PITX1, PITX2), LIM homeobox (LHX3, LHX4) or late-acting such as PROP1 (prophet of Pit-1) and POU1F1 (previously called PIT-1) [22]. *Lhx3* gene expression is detected in the developing nervous system and Rathke’s pouch at approximately E9 [6]. By E12, POU1F1 synergistically partners with PITX1 and PITX2 for the differentiation of specific pituitary-specific cell types; thyrotropes, lactotropes and somatotropes [23]. The gonadotrope and thyrotropic precursors are activated by zinc-finger transcription factor, GATA2 in response to inductive interaction of LHX3 and BMP2 signaling [20]. Terminal differentiation of the pituitary completes at approximately E17 in response to the tightly controlled temporospatial gradient expression of transcription factor [7, 24]. A simplified scheme of expression of the transcription factors is given in Fig. 3. In *Lhx3* null mutant mice, Rathke’s pouch is initially formed but then fails to grow resulting in either stillbirth or death shortly following birth, thus providing a genetic paradigm for the study of pituitary development [25]. LHX3 is known to plays an essential role in the proper development of the spinal motoneurons (which likely explains neck rotation anomalies in human with *LHX3* mutation) and is also expressed in the auditory system [4, 26, 27]. Only a small number of studies have previously reported the clinical phenotype and genetic basis of *LHX3* patients (Human Gene Mutation Database [28]). Together with two novel mutations identified in our study, there are now 16 different *LHX3* variants (Fig. 2e) in patients with CPHD3 phenotype as detailed in Table 3. The clinical phenotypes of human *LHX3* mutations encompass a varied presentation. The earliest descriptions of *LHX3* mutations were associated with panhypopituitarism without ACTH deficiency or any extrapituitary phenotypes [29]. Later, a rigid cervical spine with limited head rotation, mental retardation and MRI findings of a hypodense lesion in the pituitary was also reported in addition to CPHD [30]. Four homozygous *LHX3* mutations with CPHD, with or without limited neck rotation were identified in a cohort of 366 patients with hypopituitarism or CPHD. Hearing status in these patients was not documented to be either normal or with any hearing deficit [31]. Bilateral SNHL was first associated as an additional CPHD3 phenotype in the four patients with CPHD, severe pituitary hypoplasia, ACTH deficiency and skeletal abnormalities [32]. The occurrence of SNHL was explained by expression of *LHX3* in a pattern overlapping that of *SOX2* in the sensory epithelium of developing inner ear [32]. Later, six patients from a same genealogy with CPHD, restricted neck rotation, scoliosis and congenital hearing impairment were described to harbor a recessive novel splice acceptor site mutation [33]. More recently, a complete loss of function mutation is associated with CPHD including ACTH deficiency, short neck and SNHL [5]. Compound heterozygous *LHX3* defects in a nonconsanguineous patient with syndromic CPHD, severe scoliosis and normal intelligence and a novel homozygous mutation p.T194R were recently reported [34, 35].

**Table 3:** *LHX3* mutations in patients with CPHD3 phenotype

No.	Mutation	Amino acid change	Mutation Type	Reference	
				Authors	Journal [Reference No]
1	c.148A > T	Lys50Ter	Nonsense	Rajab A et al.	Hum Mol Genet 2008 [32]
2	c.229C > T	Arg77Ter	Nonsense	Bonfig W et al.	Eur J Pediatr 2011 [5]
3	c.347A > G	Tyr116Cys	Missense	Netchine I et al.	Nat Genet 2000 [29]
4	c.368G > A	Cys123Tyr	Missense	Sobrier M et al.	J Clin Endocrinol Metab 2012 [35]
5	c.437G > T	Cys146Phe	Missense	Ramzan K et al.	BMC Endocr Disord
6	c.466C > T	Arg156Ter	Nonsense	Ramzan K et al.	BMC Endocr Disord
7	c.581A > G	Gln194Arg	Missense	Bechtold-Dalla Pozza S et al.	Horm Res Paediatr 2012 [34]
8	c.644C > T	Ala215Val	Missense	Pfaeffle R et al.	J Clin Endocrinol Metab 2007 [31]
9	c.687G > A	Trp229Ter	Nonsense	Pfaeffle R et al.	J Clin Endocrinol Metab 2007 [31]
10	c.267-3C > G		Splicing	Sobrier M et al.	J Clin Endocrinol Metab 2012 [35]
11	c.470-2 A > G		Splicing	Kristrom B et al.	J Clin Endocrinol Metab 2009 [33]
12	c.111delT	Gly38Alafs*140	Deletion	Bhangoo A et al.	J Clin Endocrinol Metab 2006 [30]
13	c.302_303delG CinsTCCT	Gly101Valfs*78	Small indel	Pfaeffle R et al.	J Clin Endocrinol Metab 2007 [31]
14	<1.4 Mb incl. entire gene		Gross deletion	Pfaeffle R et al.	J Clin Endocrinol Metab 2007 [31]
15	23 bp E3I3-3 to E3I3 + 20		Gross deletion	Netchine I et al.	Nat Genet 2000 [29]
16	3088 bp incl. ex. 2-5		Gross deletion	Rajab A et al.	Hum Mol Genet 2008 [32]

Nucleotide and amino acid numbering are based on *LHX3*, variant 2 (also known as isoform b) and correspond to NCBI reference sequence accession number NM_014564.3 for the cDNA and NP_055379.1 for the protein. Nucleotide numbering commenced with the A of the ATG translation initiation codon as +1

A candidate-gene approach was used on the basis of documented pituitary abnormalities, restricted neck rotation and SNHL, *LHX3* gene was then sequenced. Herein, we characterize two novel mutations in three patients from two unrelated Saudi consanguineous families. The three patients presented with early infantile hypoglycemia and deficiency of all anterior pituitary hormones including ACTH. Two of the affected patients also displayed rigidity of the cervical spine and short neck with limited rotation. Clinically, there was no evidence of cardiac defects or skin manifestations as reported earlier in some reports [32, 34]. Birth length in one of our patients was slightly below the mean centile for gestational age, which supports earlier findings that IGFs are necessary for that period but the severe deficiency interacts with intrauterine longitudinal growth [33]. Hearing impairment was noticed for all three affected at the age of 2–3 years, audiologic assessment later confirmed moderate SNHL that was partially managed with hearing aids and all had receptive and expressive language delay. Perinatal mortality has been reported in other families with *LHX3* mutations; our study family 1 had a baby girl who died at an early age probably due to the similar phenotype. Both the male patients had markedly delayed pubertal development and micropenis symptom, which can thus facilitate the diagnosis in case of a male child. Short neck and neonatal hypoglycemia may be overlooked in neonatal cases, but the prolonged jaundice and hypothyroidism should lead to the diagnosis of CPHD.

The incidence of reported mutations in *LHX3* gene remains low. Clinical, hormonal and radiological work-up is very important to better determine which transcription factor should be screened. We suggest that the presence of SNHL and limited neck rotation be considered in the differential diagnosis of CPHD3 to facilitate molecular diagnosis. The identification of a *LHX3* mutation at an early age is likely to be beneficial for patients to start an appropriate replacement of hormone deficiencies, auditory testing to allow regular speech development, counseling for limitations of cervical mobility and development of scoliosis, and for continual monitoring of these patients. The importance to characterize the patients with CPHD should be emphasized to physicians and researchers to help the genetic screening of patients, and to assist genetic counseling and prenatal diagnosis.

## Abbreviations

ACTH: Adrenocorticotrophic hormone
CPHD: Combined pituitary hormone deficiency
FSH: Follicular stimulating hormone
FT4: Free thyroxine
GH: Growth hormone
IGF1: Insulin growth factor 1
IGFBP3: Insulin growth factor binding protein 3
LH: Leutinizing hormone
LIM-HD: LIM-homeodomain
MRI: Magnetic resonance imaging
PRL: Prolactin
SNHL: Sensorineural hearing loss
TSH: Thyroid stimulating hormone
Znf: Zinc-finger
